# Sociodemographic characteristic of changes in smoking patterns in rural and urban population of PURE Poland study: findings from 6-year follow up

**DOI:** 10.1186/s12889-018-6354-0

**Published:** 2019-01-03

**Authors:** Katarzyna Połtyn-Zaradna, Katarzyna Zatońska, Alicja Basiak, Barbara Sozańska, Dagmara Gaweł-Dąbrowska, Maria Wołyniec, Andrzej Szuba, Witold Zatoński

**Affiliations:** 1 Department of Social Medicine, Wroclaw Medical University, Bujwida 44, 50-345 Wrocław, Poland; 2 1st Department and Clinic of Paediatrics, Allergology and Cardiology, Wroclaw Medical University, Chałubińskiego 2a, 50-368 Wrocław, Poland; 3 Department of Internal Medicine, 4th Military Hospital in Wroclaw, Rudolfa Weigla 5, 50-981 Wrocław, Poland; 4 Department of Angiology, Wroclaw Medical University, Bartla 5, 51-618 Wrocław, Poland; 5 Health Promotion Foundation, Mszczonowska 51, 05-830 Nadarzyn, Poland

**Keywords:** PURE, Tobacco, Urban, Rural

## Abstract

**Background:**

Tobacco smoking is one of the most serious modern civilization threats. According to WHO identifying patterns of tobacco use is essential for implementing effective preventive policies. The aim of the paper was to assess changes in smoking patterns among the PURE study population over 6 years.

**Methods:**

The study sample comprised of 1784 adult participants from PURE Poland study, who were assessed at baseline (2007–2010) and then at 6-year follow-up. Participants were classified into current smokers, ex-smokers and never smokers. Smoking patterns were analyzed according to sex, age/birth cohort, place of residence (urban vs rural setting), and education level.

**Results:**

Overall, a significant decrease of 3.1% in current smokers was observed (from 20.0% in baseline to 16.9 at follow-up). However, 0.8% of never smokers and 6.2% of ex-smokers at baseline were classified as current smokers at 6-year follow-up. Despite overall decrease in percentage of current smokers in both rural and urban area, in fact significantly more ex-smokers from rural area became current smokers after 6 years. Living in the rural area was associated with nearly two-fold increase in current smoking, and almost two-fold decline in chances to quit smoking. The highest percentage of current smokers was observed in birth year cohort 1961–1979.

**Conclusion:**

Despite a small but significant decline in overall smoking rates, important differences in smoking and quitting patterns emerged between rural and urban areas, as well as sexes. A less favorable smoking patterns were observed among women, and rural populations, suggesting that these are important targets of future tobacco control interventions in Poland.

## Background

Tobacco smoking is one of the most serious modern civilization threats. This is a widespread phenomenon and brings losses to society and the economy. Tobacco consumption is responsible for 700,000 deaths each year in European Union [1]. Moreover, in Poland we can talk about a specific epidemic of tobacco dependence, which results can be seen in disability, reduced development potential, and the deaths of many thousands of people of full productivity each year [2]. Around 50% of smokers in Europe die prematurely, resulting in the loss of an average of 14 years of life [1]. Almost one in three Polish men do not live to the age of 65, and almost half of this premature mortality can be associated with much higher prevalence of smoking in Poland than in Western Europe [3].

Sales and consumption of cigarettes in Poland began to increase rapidly after World War II and reached a peak in the late 1970s [4]. A comparison of international data indicates that the consumption of cigarettes in Poland in the 1970s and 1980s was among the highest in the world, reaching about 3600 cigarettes per adult Pole. At the end of the 1980s, and especially in the 1990s, the sale of cigarettes decreased, and there was a decline in social acceptance of smoking. Comparative international studies conducted in Poland and the European Union showed that in the late 1990s the social attitude to reduce the health consequences of smoking was among the best in Europe [5]. Several millions of Poles quitted smoking and a decline in the incidence of smoking had been observed in the results of 1991 health campaign “Stop smoking with us” [6].

Earlier observed changes in smoking patterns of Poles could be attributed to a range of factors, including an initiation of education on the health consequences of smoking [7], an implementation of modern legislation [8] and the government’s antitobacco program. The latter was first implemented in 1997–2001 as a program of health and socio-economic policy leading to the reduction of tobacco consumption in Poland [9]. In years 2002–2006 more governmental programs of limiting the health consequences of smoking in Poland were introduced. In 2010 further legislation has been introduced to reduce tobacco use in public places [10]. A continuation of the program to reduce the prevalence of health consequences of smoking under the auspice of the Ministry of Health has been planned for years 2014–2018 [11]. The systemic basis for the fight against tobacco epidemic is defined in Poland by: the Law on the Protection of Health of the Consequences of the Use of Tobacco and Tobacco Products [8, 10]. It covers the most important areas of tobacco control policy, including: advertising bans, no smoking in public places, the need to place anti-tobacco warnings on packages (30% of the largest packaging plane) and the introduction of pictorial signs. These actions contributed to changes in attitudes towards smoking.

According to World Health Organization monitoring tobacco smoking prevalence and initiation of scientific research addressing determinants and consequences of smoking are essential for effective tobacco control [12]. WHO Report on the Global Tobacco Epidemics 2017 emphasizes that identifying patterns in tobacco use helps to design more targeted control policies [13].

Regular statistical surveys, economic and health analyzes provide the basis for a robust and effective fight against this threat at system level. Several studies on smoking and cessation have been carried out in Poland. For example, study carried out by TNS Poland on the order of Główny Inspektorat Sanitarny (The Chief Sanitary Inspectorate) clearly indicate a downward trend in the proportion of regular smokers - from 31% in 2011 to 24% in 2015, and an increase in the proportion of people who never smoked [14]. Downward trend is smoking prevalence can be also observed in our study, but because of its cohort design, the results are even more valuable. Few previous studies [15, 16] have shown an important differences in smoking patterns between residents of urban and rural areas in Poland.

The Prospective Urban and Rural Epidemiology Study (PURE) is an international, global cohort study comprising 21 countries and more than 150,000 participants worldwide [17]. PURE Poland is a unique in the scale of the entire Poland longitudinal cohort assessment of smoking patterns in urban population (in city of Wrocław) and rural population (surrounding Wrocław). In our previous analysis of baseline data from PURE we found greater prevalence of smoking among rural inhabitants [18]. The aim of this study was to assess of changes over 6 years in tobacco consumption among the study sample with special attention paid to sociodemographic characteristics of the population.

## Methods

All participants were assessed in accordance to the PURE project protocol, which has been described in detailed elsewhere [17, 19]. Participants were recruited and assessed at baseline between 2007 and 2010. The PURE-Poland study baseline covered a group of 2036 adults (1277 women and 758 men), including both urban (59.4%) and rural (40.6%) inhabitants of Lower Silesia in Poland. At 6-year follow up the re-contact rate was 87.6%, and comprised of 1784 participants (64.5% women). Overall, 12.4% of participants were lost to follow-up, with attrition due to deaths (4.3%), refusals (5.7%) and lack of contact with participants (2.4%). The following study presents results of 1784 participants that have partaken both in the baseline study and in the 6-year follow up. 60.3% of the participants were urban dwellers. Participants were divided into three birth-year cohorts: born before 1940 (ages 67–85 at baseline), between 1940 and 1960 (ages 47–69) and 1961–1979 (ages 30–48). In the described period the mean age increased by 7.2 years, from 54.2 ± 9.6 (min. 30 years, max. 85 years) to 61.4 ± 9.6 (min.36 years, max. 92 years). Detailed characteristics of investigated population at baseline and follow-up are presented in Table 1.

**Table 1:** Characteristics of 1784 participants of PURE Poland study population

	Total		Wrocław		Villages	
Man: n (%)	634 (35.5%)		392 (36.5%)		242 (34.1)	
Female: n (%)	1150 (64.5%)		683 (63.5%)		467 (65.9%)	
	Baseline 2007–2010	6 years study	Baseline 2007–2010	6 years study	Baseline 2007–2010	6 years study
Man Age: median (range)	53.8 (±9.9)	60.9 (±9.9)	53.9 (±9.5)	61.1 (±9.5)	53.5 (±10.4)	61.6 (±10.4)
Birth-year cohort Man						
< 1940	38 (6.0)		14 (3.6)		24 (9.9)	
1940–1960	420 (66.2)		277 (70.6)		143 (59.1)	
1961–1979	176 (27.8)		101 (25.8)		75 (31.0)	
Women Age: median (range)	54.5 (±9.4)	61.6 (±9.5)	54.5 (±8.6)	61.6 (±8.6)	54.5 (±10.6)	61.6 (±10.6)
Birth-year cohort Women						
< 1940	73 (6.3)		22 (3.2)		51 (10.9)	
1940–1960	835 (72.6)		529 (77.5)		306 (65.5)	
> 1960	242 (21.1)		132 (19.3)		110 (23.6)	
Total Age: median (range)	54.2 (±9.6)	61.3 (±9.6)	54.3 (±8.9)	61.4 (±9.0)	54.5 (±10.6)	61.3 (±10.5)
Total Birth-year cohort						
< 1940	111 (6.2)		36 (3.3)		75 (10.6)	
1940–1960	1225 (70.4)		806 (75.0)		449 (63.3)	
> 1960	418 (23.4)		233 (21.7)		185 (26.1)	
Education level						
Primary	244 (13.7%)		36 (3.3%)		208 (29.3%)	
Vocational	287 (16.1%)		70 (5.5%)		217 (30.6%)	
Secondary	704 (39.4%)		476 (44.3%)		228 (32.2%)	
Higher	546 (30.6%)		490 (45.6%)		56 (7.9%)	
Missing	3 (0.2%)		3 (0.3%)		0 (0.0%)	

Both in baseline and 6-year follow-up the history of tobacco use was assessed. The participants chose one of three possible answers: formerly used tobacco products; currently use tobacco products and never used tobacco products. Worldwide in 6-year follow-up there were some detailed criteria added to the query regarding history of smoking. In baseline regular use was defined as consuming at least one tobacco product per day. In 6-year follow-up current smokers were defined as participants who have smoked at least 100 cigarettes in their lifetime and currently smoke cigarettes everyday (daily) or some days (non-daily). Moreover, in 6-year follow-up never smokers were defined as participants who have never smoked or who have smoked fewer than 100 cigarettes in their lifetime.

Data was analyzed using chi-square and logistic regression, with the smoking status at follow-up regressed onto with sex, birth cohort, place of residence, and education. The findings were reported as Odds Ratios (ORs) with 95% Confidence Intervals (CIs). The significance level was established as *p* ≤ 0.05. Data was analyzed in Statistica 12.0.

Informed consent was obtained from all individual participants included in the study. All human studies have been reviewed by the appropriate ethics committee and have been performed in accordance with the ethical standards laid down in an appropriate version of the 1964 Declaration of Helsinki (Positive opinion of The *Bioethics Committee* of the *Wrocław Medical University* nr KB- 443/2006).

## Results

In the analyzed period a statistically significant decrease of 3.1% in number of current smokers (from 20.0% in baseline to 16.9 in follow-up) was observed. At baseline there were 20.0% current smokers, 31.7% former smokers, and 48.3% never smokers. Among the follow-up sample there were 16.9% current smokers, 34.9% ex-smokers, and 48.2% never smokers. Despite the overall decrease in percentage of current smokers, 0.8% of never smokers and 6.2% of former smokers at baseline became current smokers at 6-year follow-up. Smoking patterns were different across sexes, birth-year cohorts, places of residence and education level (Table 2).

**Table 2:** General characteristics of attitude towards tobacco smoking among 1784 participants of PURE Poland study

Characteristics	Ever smokers								Never smokers		*P*-value					
	Current smokers			Former smokers			Total									
	Baseline 2007–2010	6 years study	*P*-value	Baseline 2007–210	6 years study	*P*-value	Baseline 2007–2010	6 years study	Baseline 2007–2010	6 years study	a)	b)	c)	d)	e)	f)
Total N % (95% Cl)	356 20.0 (18.1–21.9)	302 16.9 (15.2–18.8)	0.019	566 31.7 (29.6–33.9)	622 34.9 (32.7–37.1)	0.008	922 51.7 (49.3–54.0)	924 51.8 (49.4–54.1)	862 48.3 (46.0–50.7)	860 48.2 (45.9–50.6)	–	–	0.029	0.947	–	–
Sex																
Men N % (95% Cl)	142 22.4 (19.2–25.8)	116 18.3 (15.4–21.5)	0.081	263 41.5 (37.6–45.4)	288 45.4 (41.5–49.4)	0.053	405 63.9 (60.0–67.6)	404 63.7 (59.8–67.5)	229 36.1 (32.4–40.0)	230 36.3 (32.5–40.2)	< 0.001	< 0.001	0.153	0.953	< 0.001	< 0.001
Women N % (95% Cl)	214 18.6 (16.4–21.0)	186 16.2 (14.1–18.4)	0.171	303 26.3 (23.8–29.0)	334 29.0 (26.4–31.8)	0.063	517 44.9 (42.1–47.9)	520 45.2 (42.3–48.1)	633 55.1 (52.1–57.9)	630 54.8 (51.9–57.7)			0.176	0.90		
Birth-year cohort																
< 1940 N % (95% Cl)	2 1.8 (0.2–6.4)	6 5.4 (2.0–11.4)	0.150	33 29.7 (21.4–39.1)	30 27.0 (19.0–36.3)	0.144	35 31.5 (23.0–41.0)	36 32.4 (23.9–42.0)	76 68.5 (59.0–77.0)	75 67.6 (58.0–76.1)	< 0.001	< 0.001	0.341	0.886	< 0.001	< 0.001
1940–1960 N % (95% Cl)	269 20.7 (19.2–23.8)	208 16.6 (14.6–18.7)	0.002	430 34.3 (31.6–37.0)\	479 38.2 (35.5–40.9)	0.001	690 55.0 (52.2–57.8)	687 54.8 (51.9–57.5/)	565 45.0 (42.2–47.8)	568 45.2 (42.5–48.1)			0.005	0.904		
1961–1979 N % (95% Cl)	94 22.5 (18.6–26.6)	88 21.1 (17.2–25.3)	0.675	103 24.5 (20.6–29.1)	113 27.0 (22.8–31.6)	0.492	197 47.0 (42.3–52.0)	201 48.1 (43.2–53.0)	221 52.9 (48.0–57.7)	217 51.9 (47.0–56.8)			0.697	0.080		
Place of residence																
Urban N % (95% Cl)	176 16.4 (14.2–18.7)	146 13.6 (11.6–15.8)	0.070	393 36.5 (33.7–39.5)	422 39.2 (36.3–42.2)	0.050	569 52.9 (49.9–55.9)	568 52.8 (49.9–55.9)	506 47.1 (44.1–50.1)	507 47.2 (44.1–50.2)	< 0.001	0.194	0.147	0.966	< 0.001	0.277
Rural N % (95% Cl)	180 25.5 (22.2–28.8)	156 22.0 (19.0–25.2)	0.114	173 24.4 (21.3–27.7)	200 28.2 (24.9–31.7)	0.056	353 47.8 (46.0–53.5)	356 50.2 (46.5–54.0)	356 50.2 (46.5–54.0)	353 49.8 (46.0–53.5)			0.159	0.873		
Level of Education*																
Primary N % (95% Cl)	46 18.8 (14.1–24.3)	47 19.3 (14.5–24.8)	0.908	59 24.2 (18.9–30.0)	59 24.2 (18.9–30.0)	0.938	105 43.0 (36.7–49.5)	106 43.5 (37.1–49.9)	139 57.0 (50.5–63.3)	138 56.5 (50.1–62.9)	< 0.001	< 0.001	0.993	0.927	< 0.001	< 0.001
Vocational N % (95% Cl)	84 29.3 (24.1–34.9)	68 23.7 (19.2–29.4)	0.130	79 27.5 (22.4–33.1)	95 33.1 (27.7–38.9)	0.136	163 56.8 (50.8–62.6)	163 56.8 (50.8–62.6)	124 43.2 (37.4–49.2)	124 43.2 (37.4–49.2)			0.206	1.000		
Secondary	146 20.7 (17.8–23.9)	127 18.0 (15.3–21.1)	0.200	258 36.7 (33.1–40.3)	277 39.3 (35.7–43.1)	0.151	404 57.4 (53.6–61.1)	404 57.4 (53.6–61.1)	300 42.6 (38.9–46.4)	300 42.6 (38.9–46.4)			0.343	1.000		
Higher N % (95% Cl)	80 14.7 (11.8–17.9)	60 11.0 (8.5–13.9)	0.700	169 30.9 (27.1–35.0)	190 34.8 (30.8–39.0)	0.043	249 45.6 (41.4–49.9)	250 45.8 (41.5–50.1)	297 54.4 (50.1–58.6)	296 54.2 (49.9–58.5)			0.130	0.952		

a): chi-square test comparing current smokers, former smokers and never smokers in Baseline 2007

b): chi-square test comparing ever smokers and never smokers in Baseline 2007–2010

c): chi-square test comparing current smokers, former smokers and never smokers Baseline 2007–2010 and 6 years study

d): chi-square test comparing ever smokers and never smokers Baseline 2007–2010 and 6 years study

e): chi-square test comparing current smokers, former smokers and never smokers in 6 years study

f): chi-square test comparing ever smokers and never smokers in 6 years study

*-3 participants were excluded due to lack of the information about level of education

In both, men and women, an insignificant decrease in percentage of current smokers was observed (4.1% among men from 22.4% in baseline to 18.3% in follow up, and 2.4% among women from 18.6% in baseline to 16.2% in follow up) (Table 2). More women than men, who were former smokers in the baseline had reported smoking in the follow-up (7.3% of women vs. 4.9% of men), and more women than men, who never smoked started smoking in follow-up (0.9% of women vs. 0.4% of men). However, these differences between sexes were not statistically significant (*p* > 0,05). Sex was not a significant differentiating factor of being a current smoker [OR = 1.262, Cl 0.994 to 1.602 in baseline and OR = 1.161, Cl 0.900 to 1.489 in follow up]. On the other hand, the follow up showed that sex was associated with quitting smoking. Being male increased the chance of quitting smoking 1,4-fold [OR = 1.383, Cl 1.044 to 1.830].

The age of participants significantly differentiated smoking patterns. Both in baseline and 6-year follow up, the percentage of current smokers increased along with the decrease of age of the participants (Table 2). In analyzed period, statistically significant decrease of percentage of current smokers occurred only in birth cohort 1940–1960, in which, after 6 years, the risk of being a current smoker decreased by a quarter [OR = 0.727, Cl = 0. 594 to 0.889]. Moreover, the chance of quitting smoking in the birth-year cohort 1940–1960 increased over the years 1.2-fold [OR = 1.187, Cl = 1.007 to 1.400].

Alarming increase in the percentage of current smokers was observed in the oldest group, born < 1940 year (1.8% in baseline vs. 5.4% in follow-up), but these results should be interpreted with caution, as the group numbers were very small.

In the follow up, 22.0% of rural population were current smokers, while in urban population it was only 13.6%. Both in case of urban and rural residents an insignificant decrease in percentage of current smokers was observed (Table 2). Despite overall decrease in percentage of current smokers in both rural and urban area, it is worth noticing that in fact statistically significantly more former smokers from rural area became current smokers after 6 years (12.1% in rural area vs. 3.6% in urban area)(*p* < 0.001). Rural place of residence increased the risk of being a current smoker almost 2-fold [OR = 1.738 Cl = 1.376–2.196 in baseline and OR = 1.780 Cl = 1.387–2.284 in follow-up]. The detailed analysis of smoking patterns, considering sex, birth-year cohort and place of residence has demonstrated, that both men and women living in the urban area were characterized by lower percentage of current smokers than in rural areas (Table 3).

**Table 3:** Characteristics of attitude towards tobacco smoking among 1784 participants of PURE Poland study taking into account place of residence (urban-rural)

Characteristics	Current smoker						Former smoker						Never smoker					
	Baseline 2007–2010			6 years study			Baseline 2007–2010			6 years study			Baseline 2007–2010			6 years study		
	Urban	*Rural*	*p*-values	Urban	Rural	*p*-values	Urban	Rural	*p*-values	Urban	Rural	*p*-values	Urban	Rural	*p*-values	Urban	Rural	*p*-values
Sex																		
Men	66 (16.8)	76 (31.4)	0.000	54 (13.8)	62 (25.6)	0,000	184 (46.9)	79 (32.6)	0.000	194 (49.5)	94 (38.9)	0.000	142 (36.3)	87 (36.0)	0.988	144 (36.7)	86 (35.5)	0.826
Women	110 (16.1)	104 (22.3)	0.010	92 (13.5)	93 (19.9)	0.004	209 (30.6)	94 (20.1)	0.000	228 (33.4)	107 (22.9)	0.000	364 (53.3)	269 (57.6)	0.167	363 (53.1)	267 (51.2)	0.198
Birth-year cohort																		
< 1940^a^	0 (0.00)	2 (0.03)	-	1 (0.002)	5 (0.07)		16 (0.44)	17 (0.23)	-	15 (0.42)	15 (0.20)	0.043	20 (0.56)	56 (0.74)	0.516	20 (0.56)	55 (0.73)	0.061
1940–1960	140 (17.4)	120 (26.7)	0.000	110 (16.3)	98 (21.8)	0.000	320 (39.7)	110 (24.5)	0.000	347 (43.1)	132 (29.4)	0.000	346 (42.9)	219 (48.8)	0.050	349 (43.3)	219 (48.8)	0.062
1961–1979	36 (15.4)	58 (31.3)	0.000	35 (15.0)	53 (28.6)	0.001	57 (24.5)	46 (24.8)	0.001	60 (25.8)	53 (28.6)	0.060	140 (60.1)	81 (43.9)	0.001	138 (59.2)	79 (42.8)	0.001

^a^Due to very low number of participants in the group, the data was presented in rate

In men, rural place of residence increased the risk of being a current smoker over 2-fold [OR = 2.156 Cl = 1.435–3.240 in follow up]. In women, rural place of residence increased the risk of being a current smoker 1.2-fold [OR = 1.156 Cl = 1.165–2.191 in follow-up], who simultaneously had lower chance to quit smoking than men [OR = 0.224 Cl = 0.160–0.314 vs OR = 0.556 Cl = 0.358–0.864 in follow up].

In the follow up, 11.0% of participants with higher education were current smokers, in comparison to 23.7% with vocational education. Excluding the group of people with elementary education, which was observed to have an increase of current smoker percentage (from 18.8% in baseline to 19.3% in follow up), in remaining groups a decrease in current smokers percentage was observed. All changes were not statistically significant (Table 2). The highest percentage of former smokers, who became current smokers after 6 years, was observed among participants with primary education (12.7%), then participants with secondary education (7.4%). Education with the minimum of secondary education decreased the odds of being a current smoker [secondary education: in baseline: OR = 0.632 Cl 0.463 to 0.865, in follow-up OR = 0.709 Cl 0.508 to 0.989 and in higher education: in baseline OR = 0.632 Cl 0.426 to 0.865, in follow-up OR = 0.561 Cl 0.403 to 0.780]. Furthermore, the follow up showed that higher education began to be a factor that increased the chance of quitting smoking compared to secondary education [OR = 1.459 Cl 1.015 to 2.077]. In 6-years follow-up participants with secondary education had almost 2-fold higher chance of being a former smoker than those with vocational education [OR = 1.579 Cl 1.085 to 2.230].

## Discussion

This study reports findings on changes in smoking patterns from baseline (2007–2010) [18] to 6-year follow-up in an adult cohort study in Poland (PURE Poland). The findings suggested a small, but significant decrease in the overall percentage of current smokers, which is consistent with findings from other studies [1, 15, 20, 21]. In our study, about one-sixth of the population in baseline were current smokers. Over 6-years period, percentage of current smokers decreased significantly by 3.1%. This decrease was more pronounced among men than women, in urban than in rural population and among participants born between 1940 and 1960. According to the Report of the European Commission published in March 2017, the percentage of current smokers decreased in the whole EU by 6% over the years 2006–2017 [1]. We researched only half of this period (2007–2013). Up to 2013, the percentage of current smokers in our cohort decreased accordingly to the percentage of current smokers in the EU. Similar decrease in smoking prevalence was observed in other polish studies [15, 20]. According to the Report of the European Commission 30% of Poles are current smokers. This percentage is higher than findings from our research (20.0% in baseline and 16.1% in 6-year follow up), but also researched age groups are different: EU Report takes into consideration participants above 15 years old, our cohort consists of participants above 29 years old. The different range of participant’s age may affect the discrepancy between percentages of current smokers in Poland. This speculation can be also supported by the fact, that in our study, the highest prevalence of current smokers in baseline survey was observed in the youngest birth-year cohort 1961–1979. In our findings sex was statistically significant differentiating factor of smoking patterns. According to our findings the percentage of current smokers is higher among men than women. The same tendency can be observed in mentioned studies of Sozańska et al. [15], WOBASZ studies [20], Global Burden Disease Study [22] and Report of European Comission [1]. On the contrary, in our findings men were 1.37-fold more likely to quit smoking than women, which is consistent with results obtained by Sozańska et al. [15] (Women: OR = 0.76, 95% Cl = 0.55–1.07 in town in 2012 and OR = 0.50, Cl = 0.35–0.72 in village in 2012). In our findings the percentage of former smokers both men and women increased over the years, which is a positive outcome (41.5 to 45.4% in men and 26.3 to 29.1% in women). Participants of birth-year cohort 1940–1960 were more likely to quit smoking than any other birth-year cohort, in fact we observed the highest decrease of the percentage of current smokers in this birth-year cohort (20.7% in baseline vs. 16.6% in 6-year follow-up), which is consistent with findings from EU Report [1], which stated that 30% of people between 40 and 54 years old quit smoking. On the other hand, we observed statistically insignificant decrease of current smokers in birth cohort 1961–1979, which is inconsistent with findings from EU Report, where most smokers quitted habit in middle age: 25–39 years (38%) and 40–54 years (30%).

In our results, rural dwellers smoked more than urban dwellers, both in baseline and 6-year follow up, but the percentage decreased over time in both places of residence (from 16.4 to 13.6% in urban dwellers and from 25.5 to 21.9% in rural dwellers). The rural place of residence increased the risk of being current smoker nearly two fold in the 6-year follow up and this risk increased since the baseline results. In the study of Sozańska et al. [15], rural place of residence increased the risk of being a current smoker almost three-fold. On the contrary, findings from Polish arm of Global Tobacco Survey (GATS) conducted between 2009 and 2010 suggested that in fact urban dwellers were more likely to smoke than rural dwellers back then [16]. GATS study was performed nationwide and study of Sozańska et al. [15] in the same region as our study, we speculate that this fact could explain the results. On the other hand, however, a higher rate of current smokers among rural dwellers can be observed also worldwide. According to the data (2007–2014) obtained within Nations Survey of Drug Use and Health – nationally representative study of participants aged 12 years or older in United States – the percentage of currents smokers in rural areas is much higher than in urban areas [23]. According to our results, having at least secondary education decreased the odds of being a current smoker. Moreover, in 6-year follow-up, from secondary education and above the chance to quit smoking is significantly higher. As stated in EU report, smoking prevalence was greater among those who finished their education earlier [1]. Conforming to above results, data obtained within National Health Interview Survey conducted in 2015 in United States among noninstitutionalized inhabitants, show that the percentage of current smokers decreased proportionally with increasing level of education (24.2% of participants with primary education were current smokers in comparison to 3.6% of participants with graduate degree) [24]. Those results suggest that antitobacco programs should be directed especially to less educated people. The identification of psychosocial factors affecting tobacco smoking in our cohort, but also more widely among women and rural populations, requires further research.

Some of the limitations of the study should be taken into account. The study relied on self-reported smoking status, with no biochemical verification. International study comparing biochemically assessed and self-reported smoking rates showed that the results based on self- reporting may underestimate smoking prevalence, as it was noted in Poland at the level of 4%, and this result was higher than in other countries like England and the USA [25]. The additional limitation of the study may be the omission of some sociological factors in the analysis, such as income or employment that have been associated with tobacco smoking [26]. On the other hand, detailed analysis of level of education (primary, vocational, secondary, higher education) can show the sociological factors that influence the attitude toward smoking similarly to income or job. A limited number of participants in individual groups influence the precision of analysis. On the other hand, strength of the study is that it is a cohort study with use of the same methodology in every follow-up. Attrition of study participants from baseline compares favorably with other such studies with long-term follow-up [27, 28]. More detailed criteria for current smokers, former smokers and never smokers introduced in 6-year follow-up haven’t significantly altered our results. Only 1.3% of participants changed the smoking status to never smoker (including 1.0% of participants, who declared in baseline to be a former smoker and 0.3%, who declared themselves as current smoker). Due to enrollment rate and study logistics, both baseline and follow-up assessments, were extended over almost 3 years, which means that at least in principle participants could be exposed to different policy or health promotion campaigns. However, we were not aware of any campaigns that could differently affect participants.

## Conclusion

According to our observations, rural place of residence increases the risk of being a current smoker, and that tendency has not changed over the years. It is essential to conduct further research identifying psychosocial factors, e.g. awareness of health threat of tobacco smoking or reasons behind initiating, quitting and returning to smoking especially among women in rural areas. Identification of those factors will allow to design more individualized and targeted health promotion programs. Additionally, despite the overall decrease in percentage of current smokers over the years, it is essential to note, that some of the ex-smokers return to smoking during this period. Changes in attitudes towards tobacco smoking observed over 6 years give strong rationale for implementing comprehensive, evidence-based intervention among current smokers in our cohort, but also in other rural areas, and especially among women.

## Abbreviations

EU: European Union
GATS: Global Tobacco Survey
PURE: Prospective Urban and Rural Epidemiology Study
WHO: World Health Organization
WOBASZ: Multi*-*environment National Research of Public Health
